# Differences in provider approach to initiating and titrating guideline directed medical therapy in heart failure with reduced ejection fraction

**DOI:** 10.1186/s12872-024-03911-1

**Published:** 2024-05-11

**Authors:** David J. Cordwin, Jessica Guidi, Lana Alhashimi, Scott L. Hummel, Todd M. Koelling, Michael P. Dorsch

**Affiliations:** 1 College of Pharmacy, University of Michigan, Ann Arbor, MI USA; 2 Medical School, University of Michigan, Ann Arbor, MI USA; 3 Frankel Cardiovascular Center, University of Michigan, Ann Arbor, MI USA; 4 VA Ann Arbor Health System, Ann Arbor, MI USA

**Keywords:** Guideline-directed medical therapy (GDMT), Heart failure with reduced ejection fraction (HFrEF), Evidence-based therapy, Medications

## Abstract

**Background:**

Despite the strong evidence supporting guideline-directed medical therapy (GDMT) in patients with heart failure with reduced ejection fraction (HFrEF), prescription rates in clinical practice are still lacking.

**Methods:**

A survey containing 20 clinical vignettes of patients with HFrEF was answered by a national sample of 127 cardiologists and 68 internal/family medicine physicians. Each vignette had 4–5 options for adjusting GDMT and the option to make no medication changes. Survey respondents could only select one option. For analysis, responses were dichotomized to the answer of interest.

**Results:**

Cardiologists were more likely to make GDMT changes than general medicine physicians (91.8% vs. 82.0%; OR 1.84 [1.07–3.19]; *p* = 0.020). Cardiologists were more likely to initiate beta-blockers (46.3% vs. 32.0%; OR 2.38 [1.18–4.81], *p* = 0.016), angiotensin receptor blocker/neprilysin inhibitor (ARNI) (63.8% vs. 48.1%; OR 1.76 [1.01–3.09], *p* = 0.047), and hydralazine and isosorbide dinitrate (HYD/ISDN) (38.2% vs. 23.7%; OR 2.47 [1.48–4.12], *p* < 0.001) compared to general medicine physicians. No differences were found in initiating angiotensin-converting enzyme inhibitor/angiotensin receptor blocker (ACEi/ARBs), initiating mineralocorticoid receptor antagonist (MRA), sodium-glucose transporter protein 2 (SGLT2) inhibitors, digoxin, or ivabradine.

**Conclusions:**

Our results demonstrate cardiologists were more likely to adjust GDMT than general medicine physicians. Future focus on improving GDMT prescribing should target providers other than cardiologists to improve care in patients with HFrEF.

**Supplementary Information:**

The online version contains supplementary material available at 10.1186/s12872-024-03911-1.

## Background

Heart failure (HF) is a leading cause of hospitalizations, morbidity, and mortality. The prevalence of HF is projected to rise by 46% from 2012 to 2030, affecting more than 8 million people in the US [1]. HF hospitalizations are costly and continue to increase, with the risk of rehospitalization after discharge persisting over time [2]. Hospital readmissions due to HF can be attributed to factors including the progression of the illness, distressing symptoms, issues related to diet, medication adherence, and health system failures, such as suboptimal healthcare delivery [3]. Lack of medication optimization may be a strong contributing factor to HF rehospitalizations. Guideline-directed medical therapy (GDMT) has been proven to reduce morbidity, mortality, and hospitalizations in patients with heart failure with reduced ejection fraction (HFrEF) [4–8]. 

Despite the evidence, medication use and dosing are not always consistent with optimal GDMT in clinical practice [4]. The Change the Management of Patients with Heart Failure (CHAMP-HF) registry studied GDMT use in heart failure with reduced ejection fraction (HFrEF) among 150 US primary care and cardiology practices. In CHAMP-HF, only 73% of patients were treated with (angiotensin-converting enzyme inhibitor) ACEi, 67% were treated with beta-blockers, and 33% were treated with mineralocorticoid receptor antagonist (MRA), with less than 30% of patients being prescribed the target dose for each medication [9]. Previous studies suggest between-specialty differences between prescribers in GDMT optimization. One study comparing prescribing patterns between primary care physicians and cardiologists reported that cardiologists more commonly utilized beta-blockers and ACEI in HF treatment [10]. Another study found that community hospital patients cared for by an attending cardiologist compared to a non-cardiologist attending were more likely to be discharged on an ACEI/angiotensin receptor blocker (ARB) and a beta-blocker and had lower readmission rates [11]. These studies were completed over 20 years ago and may not reflect current practices in treating HFrEF. A better understanding of provider priorities in prescribing GDMT is needed to facilitate improvements in HFrEF care.

This study aimed to survey cardiologists and general medicine physicians on their ideal and current approach to GDMT prescribing using clinical vignettes and to examine what factors may influence any differences between the two groups.

## Methods

### Study design

This was a prospective survey of physicians about GDMT prescribing. We recruited a group of cardiologists, heart failure specialists, and general medicine physicians through Dynata. This large data firm maintains survey participant panels to answer clinical scenario questions in an online survey. The anonymous online survey was developed using Qualtrics. We used quotas to stop the survey once we reached 100 cardiologists, 50 heart failure specialists, and 50 general medicine practitioners, totaling 200 practitioners in the United States. Among cardiologists, the sampling percentage of heart failure specialists was lower than expected. This led to a higher number of cardiologists sampled in the final survey and a lower than the preferred number of HF specialists. The University of Michigan IRB reviewed the study and determined it to be exempt.

### Survey instrument

The survey consisted of 20 separate clinical vignettes describing synthetic patients. Each vignette included the patient’s age, sex, race, New York Heart Association (NYHA) class, volume status, current medication regimen, and vitals. We then presented five or six options for the providers to choose the next medication change. An example vignette from the survey is shown in Table 1.We also collected general information about the respondents, including sex, the number of patients with heart failure they care for per week, years of practice caring for patients with heart failure, and confidence in prescribing and titrating heart failure therapies. The study team initially designed the survey (MPD, TMK, SLH, JG). Heart failure cardiologists and cardiology pharmacists at the University of Michigan reviewed and commented on the questions and design of the survey. Revisions were then implemented in the survey iteratively, and Dynata distributed the final survey from December 2020 to January 2021. The final survey can be viewed in additional file 1. The questions were presented randomly to get an even number of responses per question in case participants did not complete the full survey. Before the survey dissemination, the authors created a table classifying each question for statistical analysis. For instance, some questions were designed to understand the approach to a medically naive patient, while others were designed to gain insight into which agents are titrated first. The table can be found in Table S1.

**Table 1:** Example survey vignette

Q3 A 48-year-old African American male presents to clinic with symptoms of heart failure. In the past month, he endorses NYHA class II symptoms and is not volume overloaded on physical exam. He also has a past medical history of hypertension. Medications include carvedilol 12.5 mg twice daily and lisinopril 20 mg once daily. Blood pressure is 105/65 mmHg and heart rate is 65 bpm. Labs are Na 142 mEq/L, K 4.0 mEq/L, Cr 0.8 mg/dL. Echocardiography reveals a left ventricular ejection fraction of 30%.
Increase carvedilol (1)
Add spironolactone (2)
Switch lisinopril to sacubitril-valsartan (3)
Add hydralazine and isosorbide dinitrate (4)
Add dapagliflozin (5)
Do not make any of these medication changes (6)

### Statistical analysis

*Cardiologists and heart failure specialists were grouped and compared to internal medicine physicians for analysis*. The responses to the questions were dichotomized to the research question of interest. For example, questions assessing medical naive patients were dichotomized to the answer “initiate a beta-blocker” versus any answer not initiating a beta-blocker. Generalized linear mixed-effect models with a binomial distribution assessed the statistical differences. Statistical analysis was performed with R Studio Software, version 4.0. The glmer function from the lme4 package was used to conduct the mixed effect models. Participants and the question identifier were treated as random effects. Provider type, years treating patients with heart failure, number of patients with heart failure seen per week, and confidence in adjusting HF regimens were used as fixed effects. A backward stepwise regression process was used to identify the most appropriate model for each analysis because each variable may affect the provider’s answer differently. The most complex model with all four variables was used as the initial model. The second model removed the variable with the highest p-value. ANOVA was then used to compare the two mixed models. If the p-value was less than 0.05, the model with more variables was used as the final model for that analysis. If the p-value did not reach significance, the highest p-value variable was removed and compared with the previous model. This process was repeated until the ANOVA p-value reached significance, or only one fixed effect variable remained. Variables were removed if they caused singularity in the model.

## Results

A total of 195 respondents across the United States answered at least one question in the survey. See Figure S1 for the geographic distribution of survey participants. Across all questions, there were 1268 responses from general medicine physicians and 2409 responses from cardiologists, which equated to 18.8 questions answered per respondent. The cohort characteristics are described in Table 2. 20% of the respondents were female, the average number of years caring for patients with heart failure was 18.9 years, and the average confidence for managing heart failure medications was 8.4 on a 10-point scale.

**Table 2:** Baseline characteristics

	Cardiologist (*N* = 127)	General Medicine (*N* = 68)	Overall (*N* = 195)
**Gender**			
Female	23 (18.1%)	16 (23.5%)	39 (20.0%)
Male	104 (81.9%)	52 (76.5%)	156 (80.0%)
**Heart Failure patients per week**			
0–5	6 (4.7%)	20 (29.4%)	26 (13.3%)
6–10	16 (12.6%)	17 (25.0%)	33 (16.9%)
11–15	13 (10.2%)	7 (10.3%)	20 (10.3%)
16–20	34 (26.8%)	13 (19.1%)	47 (24.1%)
21–25	18 (14.2%)	3 (4.4%)	21 (10.8%)
> 25	40 (31.5%)	8 (11.8%)	48 (24.6%)
**Years treating patients with Heart Failure**			
Mean (SD)	17.5 (9.14)	21.6 (9.34)	18.9 (9.39)
Median [Q1, Q3]	16 [10, 23]	20 [15.8, 29]	18 [12, 25.5]
**Confidence**			
Mean (SD)	8.95 (1.28)	7.35 (2.23)	8.39 (1.84)
Median [Q1, Q3]	9 [8, 10]	8 [7, 9]	9 [8, 10]

### Any medication change

For each scenario in the survey, the respondents were provided an option to defer making changes to the patient’s medication regimen. The final model for assessing changing the medication regimen included provider type and confidence as fixed effects and the participant and question as random effects. Cardiologists selected to change medication therapy in 91.8% (2211/2409) of cases compared to 82.0% (1040/1268) of cases with general medicine physicians (OR 1.84 [1.07–3.19], *p* = 0.020). Confidence in managing heart failure medications was also significantly predicted whether the provider would or would not make changes to GDMT. Providers with higher confidence were more likely to adjust medication therapy than those with lower confidence (OR 1.20 [1.04–1.37], *p* = 0.007).

### Medication initiation

**Fig. 1:**
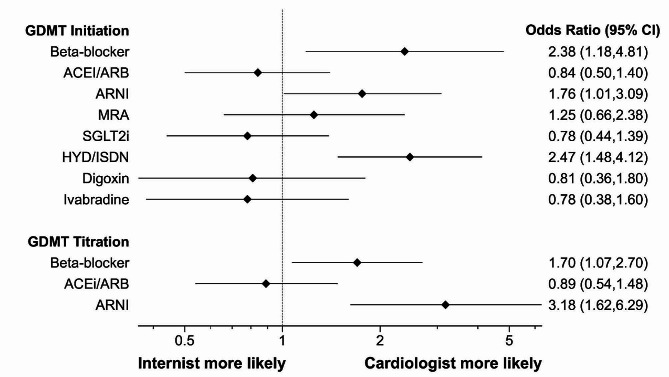
Odds ratios for initiating and titrating GDMT based on provider type. The odds ratio of initiating or titrating GDMT for cardiologists compared to general medicine physicians, controlling for baseline differences between the two groups. The diamond points represent the odds ratio and the lines represent the 95% confidence interval. Abbreviations as listed in Abbreviations List

For medically naive patients, only provider type remained in the final model after backward selection. Cardiologists were more likely to initiate beta-blockers than general medicine physicians (46.3% (112/242) vs. 32.0% (40/125); OR 2.38 [1.18–4.81], *p* = 0.016). For patients already on a beta-blocker, there was no difference between cardiologists and general medicine physicians for initiating an ACEi/ARB (35.5% (86/242) vs. 29.9% (38/127); OR 0.84 [0.5–1.40], *p* = 0.505). In patients on beta-blockers and ACEi/ARBs, there was no difference between cardiologists and general medicine physicians initiating an MRA (13.9% (50/361) vs. 11.5% (22/191); OR 1.25 [0.66–2.38], *p* = 0.495). When analyzing the initiation of angiotensin receptor blocker neprilysin inhibitor (ARNI), provider type, confidence, and years in HF practice were included in the model after backward selection. Cardiologists were more likely to initiate an ARNI than general medicine physicians (63.8% (460/721) vs. 48.1% (182/378); OR 1.76 [1.01–3.09], *p* = 0.047). Higher confidence in treating patients with heart failure and years treating patients with heart failure also increased the odds of initiating ARNI (OR 1.37 [1.17–1.60], p = < 0.001 and OR 1.03 [1.00-1.06], *p* = 0.039, respectively). There was no difference between cardiologists and general medicine physicians in initiating a sodium-glucose transporter protein 2 inhibitor (SGLT2i) (13.3% (112/840) vs. 15.6% (69/443); OR 0.78 [0.44–1.38], *p* = 0.397). Only provider type was kept in the final model when analyzing the initiation of hydralazine and isosorbide dinitrate (HYD/ISDN). Cardiologists were more likely to initiate HYD/ISDN compared to general medicine physicians (38.2% (139/364) vs. 23.7% (45/190); OR 2.47 [1.48–4.12], *p* < 0.001). There was no difference in the odds of initiating digoxin or ivabradine (20.7% (50/241) vs. 18.5% (24/130); OR 0.81 [0.36–1.80], *p* = 0.599 and 24.6% (89/362) vs. 22.3% (43/193) OR 0.78 [0.38–1.60], *p* = 0.499). The odds ratios comparing general medicine physicians to cardiologists are represented in Fig. 1.

### GDMT Titration

In the questions that assessed GDMT titration, cardiologists were more likely to titrate beta-blockers compared to general medicine physicians (22.0% (212/962) vs. 16.4% (83/506); OR 1.70 [1.07–2.70]; *p* = 0.024). Cardiologists were also more likely to titrate ARNI doses (55.3% (135/244) vs. 34.1% (43/126); OR 3.18 [1.62–6.29], *p* < 0.001). However, there was no difference between cardiologists and general medicine physicians when adjusting ACE/ARB doses (14.7% (123/838) vs. 15.1% (67/444); OR 0.89 [0.54–1.48]; *p* = 0.663). After backward selection, only provider type remained in all models assessing GDMT titration.

## Discussion

We utilized a survey with 20 clinical vignettes to evaluate how cardiologists and general medicine physicians would initiate or adjust the dosing of GDMT in hypothetical scenarios of patients with HFrEF. The providers who responded to the survey were highly experienced in caring for patients with HFrEF, with both groups reporting many years of caring for patients with HF every week. Unsurprisingly, cardiologists reported taking care of more heart failure patients and higher confidence in the adjustment of GDMT. However, the general medicine physicians who responded still reported relatively high confidence in medication management.

Providers were allowed to make no changes to GDMT for each clinical vignette. Notably, we found that general medicine physicians were about twice as likely as cardiologists to make no changes to medication (18% vs. 8.2%). Given that these were hypothetical patient scenarios, this is likely an overestimation of providers’ likelihood of adjusting GDMT in the “real world.” It is well established that many patients with HFrEF are not on optimal GDMT [9]. This gap between evidence and practice is likely multifactorial, including patients’ physiological limitations to tolerate GDMT, patient willingness to adjust medication, and providers’ therapeutic inertia, particularly when patients appear to have stable heart failure symptoms [4, 12]. In our study, lower provider confidence in adjusting GDMT resulted in a higher tendency to opt for no medication adjustment. This suggests that a physician’s self-perceived knowledge of GDMT management can impact whether a patient is placed on optimal therapy for heart failure. As the armamentarium of GDMT grows with more novel therapies, such as ARNIs and SGLT2 inhibitors, and guidelines evolve rapidly to reflect these beneficial drugs, the lack of provider confidence in managing complex GDMT regimens may continue to grow and impact optimal therapy for heart failure patients. The nuance in modern HFrEF pharmacotherapy may particularly affect general medicine physicians who may prescribe these medications less frequently for their patients with HFrEF.

Our survey study also identified several interesting findings regarding the types of medications that were initiated/titrated for patients with HFrEF. It should be noted that survey respondents only picked a single option out of several potentially reasonable responses for any given clinical scenario. First, cardiologists were more likely to opt for a beta blocker as an initial drug in medically naive patients than general medicine physicians. Cardiologists were more likely to uptitrate this medication class for patients already prescribed a beta blocker than general medicine physicians. It is notable, however, that both groups increased the dose of beta blockers less than 25% of the time when given the option, which is relevant given the dose-dependent effect on patient outcomes of this medication class [4, 13]. Next, cardiologists were significantly more likely to initiate an ARNI, which is perhaps unsurprising given its relative novelty in the guidelines, though about half of general medicine physicians responded with ARNI initiation, which is encouraging that this beneficial medication is gaining traction in the field of GDMT. On the other hand, SGLT2 inhibitors were infrequently initiated in both groups of providers, which may reflect its current underuse or providers’ preference for initiating/titrating other medications before starting an SGLT2i.

General medicine physicians play an important role in managing patients with HFrEF, either as the primary driver of GDMT management or with a cardiologist. It is important to understand how medication prescribing patterns differ between these physicians. While some studies have shown that primary care providers manage patients with HFrEF according to current evidence [14, 15], others have found that GDMT adherence is lower in patients with HFrEF cared for by general medicine physicians than cardiologists [16, 17]. However, It should be noted that the complexity and severity of heart failure seen in primary care is likely different than in patients managed by a cardiologist. One of the benefits of our study that addresses this issue is that we compared primary care and cardiologist responses to identical clinical vignettes. The findings of our survey study suggest that there may be more “missed opportunities” for medication titration in patients with HFrEF managed by primary care, as this group was more likely not to adjust medications despite patients being at sub-target doses of GDMT. In particular, initiating and titrating beta-blockers and using more novel therapies, such as ARNIs and SGLT2i, are potential areas of quality improvement and provider education within general medicine.

The differences between cardiologists’ and general medicine physicians’ GDMT prescribing are likely multifactorial, including training and education, familiarity with recent trials and guidelines, and pressure for general medicine physicians to care for patients quickly. Medication optimization is difficult even in a trial setting [4], let alone a busy primary care clinic. Clinical decision support (CDS) systems, such as treatment algorithms embedded into electronic health records, could be a future solution to closing the gap between cardiologists and general medicine physicians. Such tools could provide recommendations based on patient data for the clinician to consider and potentially help increase confidence in treating heart failure over time.

Clinical inertia is described as the lack of treatment intensification for a patient not currently at the evidence-based goals for care (O’Connor et al., 2008). Clinical inertia largely occurs in chronic diseases, with one of the most important factors contributing to clinical inertia being physician-related factors (Verhestarten et al., 2021). This lack of treatment intensification may be strongest for patients with longstanding HFrEF since some physicians may feel the patient is doing fine and may not need to change medication. Prior research supports that the efficacy and safety of newer therapies would provide reassurance in initiating new medications in these patients (Khan et al., 2020).

Our study has several limitations. Surveys are subject to self-selection bias; in the case of our study, providers who opted to respond to the survey are likely more self-assured with the management of GDMT in patients with HFrEF, as reflected in the baseline confidence of the respondents. The respondents were also clinicians with many years of experience treating patients with HF. Therefore, our survey findings may not generalize to all general medicine physicians and cardiologists, especially those just entering clinical practice. While new clinicians may be more familiar with recent data, they could have less confidence due to their limited experience. Another limitation of the study would be that no data was collected on the practice setting for each physician. Additionally, our study assessed providers’ self-reported actions to hypothetical scenarios rather than measuring true prescribing behaviors for actual patient encounters where other factors often impact decision-making regarding medications, such as medication cost, pill burden, need for lab and hemodynamic monitoring, and patient willingness to adjust medications.The survey also requires physicians to select a single answer, even though physicians may choose to make more than one change for a patient in certain situations. A combination of these limitations likely explains why medication optimization appeared higher in our study and therapeutic inertia lower amongst both groups of providers as compared to “real world” data on GDMT adherence in patients with HFrEF [9]. 

## Conclusions

The results of this survey show that cardiologists were more likely to adjust GDMT compared to general medicine physicians. Specific differences included initiation and titration of beta-blockers and ARNI. Cardiologists were more likely to initiate HYD/ISDN than general medicine physicians. Future focus on improving GDMT prescribing should target providers outside of cardiology to improve care in patients with HFrEF.

### Electronic supplementary material

Below is the link to the electronic supplementary material.

Supplementary Material 1

Supplementary Material 2

Supplementary Material 3

## Data Availability

The datasets generated during and/or analyzed during the current study are available from the corresponding author upon reasonable request.

## Abbreviations

ACEi: Angiotensin-Converting Enzyme Inhibitor
ARB: Angiotensin Receptor Blocker
ARNI: Angiotensin Receptor Blocker/Neprilysin Inhibitor
CDS: Clinical Decision Support
CHAMP-HF: Change Management of Patients with Heart Failure
GDMT: Guideline-Directed Medical Therapy
HF: Heart Failure
HFrEF: Heart Failure with Ejection Fraction
HYD: Hydralazine
ISDN: Isosorbide Dinitrate
MRA: Mineralocorticoid Receptor Antagonist
NYHA: New York Heart Association
SGLT2: Sodium-Glucose Transporter Protein 2
SGLT2i: Sodium-Glucose Transporter Protein 2 Inhibitor

## References

[1] Tsao CW, Aday AW, Almarzooq ZI (2022). Heart Disease and Stroke Statistics—2022 update: a Report from the American Heart Association. Circulation.

[2] Jencks SF, Williams MV, Coleman EA (2009). Rehospitalizations among patients in the Medicare fee-for-service program. N Engl J Med.

[3] Retrum JH, Boggs J, Hersh A (2013). Patient-identified factors related to heart failure readmissions. Circ Cardiovasc Qual Outcomes.

[4] Fiuzat M, Ezekowitz J, Alemayehu W (2020). Assessment of limitations to optimization of Guideline-Directed Medical Therapy in Heart failure from the GUIDE-IT Trial: a secondary analysis of a Randomized Clinical Trial. JAMA Cardiol.

[5] Yancy CW, Jessup M, Bozkurt B (2017). 2017 ACC/AHA/HFSA focused update of the 2013 ACCF/AHA Guideline for the management of Heart failure: a report of the American College of Cardiology/American Heart Association Task Force on Clinical Practice guidelines and the Heart Failure Society of America. J Am Coll Cardiol.

[6] Investigators SOLVD, Yusuf S, Pitt B, Davis CE, Hood WB, Cohn JN (1991). Effect of enalapril on survival in patients with reduced left ventricular ejection fractions and congestive heart failure. N Engl J Med.

[7] Effect of metoprolol CR/XL in chronic heart failure (1999). Metoprolol CR/XL Randomised intervention trial in congestive heart failure (MERIT-HF). Lancet.

[8] Pitt B, Zannad F, Remme WJ (1999). The effect of spironolactone on morbidity and mortality in patients with severe heart failure. Randomized aldactone evaluation study investigators. N Engl J Med.

[9] Greene SJ, Butler J, Albert NM (2018). Medical therapy for heart failure with reduced ejection fraction: the CHAMP-HF Registry. J Am Coll Cardiol.

[10] Edep ME, Shah NB, Tateo IM, Massie BM (1997). Differences between primary care physicians and cardiologists in management of congestive heart failure: relation to practice guidelines. J Am Coll Cardiol.

[11] Philbin EF, Weil HF, Erb TA, Jenkins PL (1999). Cardiology or primary care for heart failure in the community setting: process of care and clinical outcomes. Chest.

[12] Felker GM, Anstrom KJ, Adams KF (2017). Effect of natriuretic peptide-guided therapy on hospitalization or Cardiovascular Mortality in high-risk patients with heart failure and reduced ejection fraction: a Randomized Clinical Trial. JAMA.

[13] Fiuzat M, Wojdyla D, Pina I, Adams K, Whellan D, O’Connor CM (2016). Heart rate or Beta-blocker dose? Association with outcomes in Ambulatory Heart failure patients with systolic dysfunction: results from the HF-ACTION Trial. JACC: Heart Fail.

[14] Hirt MN, Muttardi A, Helms TM, van den Bussche H, Eschenhagen T (2016). General practitioners’ adherence to chronic heart failure guidelines regarding medication: the GP-HF study. Clin Res Cardiol.

[15] Luttik MLA, Jaarsma T, van Geel PP (2014). Long-term follow-up in optimally treated and stable heart failure patients: primary care vs. heart failure clinic. Results of the COACH-2 study. Eur J Heart Fail.

[16] Vaillant-Roussel H, Pereira B, Gibot-Boeuf S (2018). How are patients with heart failure treated in primary care?. Int J Clin Pharmacol Ther.

[17] Rutten FH, Grobbee DE, Hoes AW (2003). Differences between general practitioners and cardiologists in diagnosis and management of heart failure: a survey in every-day practice. Eur J Heart Fail.

