# Supplementary material for: Differences in provider approach to initiating and titrating guideline directed medical therapy in heart failure with reduced ejection fraction
Source: BMC Cardiovasc Disord. 2024 May 11;24:247. doi: 10.1186/s12872-024-03911-1 (PMC11087241; doi:10.1186/s12872-024-03911-1)
Supplement: Supplementary file 1 — Supplementary Material 1 [file 12872_2024_3911_MOESM1_ESM.pdf]

Additional file 1 - Survey Questions

Q1 A 65-year-old Caucasian male presents to clinic with symptoms of heart failure. In the past month, he endorses NYHA class III symptoms and is not volume overloaded on physical exam. He also has a past medical history of coronary artery disease, type 2 diabetes, and hypertension. Medications include metoprolol succinate 100mg once daily, lisinopril 20mg once daily, furosemide 40mg twice daily, aspirin 81mg once daily, atorvastatin 40mg once daily, dapagliflozin 10mg daily, and metformin 500mg twice daily. Blood pressure is 124/82 mmHg and heart rate is 75 bpm. Labs are Na 140 mEq/L, K 3.5 mEq/L, Cr 1.5 mg/dL. Echocardiography reveals a left ventricular ejection fraction of 25%.

Increase metoprolol succinate (1)

Add spironolactone (2)

Increase lisinopril (3)

Switch lisinopril to sacubitril-valsartan (4)

Do not make any of these medication changes (5)

Q2 A 55-year-old African American female presents to clinic with symptoms of heart failure. In the past month, she endorses NYHA class III symptoms and is not volume overloaded on physical exam. She also has a past medical history of coronary artery disease and hypertension. Medications include metoprolol succinate 100mg once daily, lisinopril 20mg once daily, spironolactone 25mg once daily, furosemide 80mg once daily, aspirin 81mg once daily, dapagliflozin 10mg daily, and rosuvastatin 20mg once daily. Blood pressure is 116/78 mmHg and heart rate is 78 bpm. Labs are Na 145 mEq/L, K 3.8 mEq/L, Cr 1.2 mg/dL. Echocardiography reveals a left ventricular ejection fraction of 20%.

Increase metoprolol succinate (1)

Add ivabradine (2)

Switch lisinopril to sacubitril-valsartan (3)

Add hydralazine and isosorbide dinitrate (4)

Do not make any of these medication changes (5)

Q3 A 48-year-old African American male presents to clinic with symptoms of heart failure. In the past month, he endorses NYHA class II symptoms and is not volume overloaded on physical exam. He also has a past medical history of hypertension. Medications include carvedilol 12.5mg twice daily and lisinopril 20mg once daily. Blood pressure is 105/65 mmHg and heart rate is 65 bpm. Labs are Na 142 mEq/L, K 4.0 mEq/L, Cr 0.8 mg/dL. Echocardiography reveals a left ventricular ejection fraction of 30%.

Increase carvedilol (1)

Add spironolactone (2)

Switch lisinopril to sacubitril-valsartan (3)

Add hydralazine and isosorbide dinitrate (4)

Add dapagliflozin (5)

Do not make any of these medication changes (6)

Q4 A 68-year-old Caucasian female presents to clinic with symptoms of heart failure. In the past month, she endorses NYHA class II symptoms and is not volume overloaded on physical exam. She also has a past medical history of coronary artery disease, type 2 diabetes, and hypertension. Medications include carvedilol 6.25mg twice daily, lisinopril 5mg once daily, spironolactone 12.5mg once daily, aspirin 81mg once daily, atorvastatin 80mg once daily, dapagliflozin 10mg daily, and metformin 750mg twice daily. Blood pressure is 136/65 mmHg and heart rate is 78 bpm. Labs are Na 138 mEq/L, K 4.5 mEq/L, Cr 1.4 mg/dL. Echocardiography reveals a left ventricular ejection fraction of 25%.

Increase carvedilol (1)

Increase spironolactone (2)

Increase lisinopril (3)

Add hydralazine and isosorbide dinitrate (4)

Do not make any of these medication changes (5)

Q5 A 62-year-old Caucasian male presents to clinic with symptoms of heart failure. In the past month he endorses, NYHA class II symptoms and is not volume overloaded on physical exam. He also has a past medical history of coronary artery disease and hypertension. Medications include carvedilol 25mg twice daily, lisinopril 10mg once daily, spironolactone 12.5mg once daily, dapagliflozin 10mg daily, aspirin 81mg once daily, and rosuvastatin 10mg once daily. Blood pressure is 126/76 mmHg and heart rate is 78 bpm. Labs are Na 140 mEq/L, K 4.2 mEq/L, Cr 1.2 mg/dL. Echocardiography reveals a left ventricular ejection fraction of 30%.

Increase carvedilol (1)

Increase spironolactone (2)

Increase lisinopril (3)

Switch lisinopril to sacubitril-valsartan (4)

Do not make any of these medication changes (5)

Q6 A 48-year-old African American female with a history of non-ischemic cardiomyopathy presents to clinic for follow up. In the past month she endorses NYHA class I symptoms and is not volume overloaded on physical exam. Medications include carvedilol 25mg twice daily and losartan 25mg once daily. Blood pressure is 128/74 mmHg and heart rate is 64 bpm. Labs are

Na 138 mEq/L, K 3.9 mEq/L, Cr 0.9 mg/dL. Echocardiography reveals a left ventricular ejection fraction of 25%.

Increase carvedilol (1)

Add spironolactone (2)

Increase losartan (3)

Add hydralazine and isosorbide dinitrate (4)

Add dapagloflozin (5)

Do not make any of these medication changes (6)

Q7 A 70-year-old Caucasian male presents to clinic with symptoms of heart failure. In the past month, he endorses NYHA class II symptoms and is not volume overloaded on physical exam. He also has a past medical history of coronary artery disease and hypertension. Medications include metoprolol succinate 200mg once daily, losartan 50mg once daily, spironolactone 25mg once daily, aspirin 81mg once daily, and atorvastatin 40mg once daily. Blood pressure is 136/80 mmHg and heart rate is 58 bpm. Labs are Na 134 mEq/L, K 4.0 mEq/L, Cr 1.3 mg/dL. Echocardiography reveals a left ventricular ejection fraction of 20%.

Add dapagliflozin (1)

Increase losartan (2)

Increase spironolactone (3)

Switch losartan to sacubitril-valsartan (4)

Do not make any of these medication changes (5)

Q8 A 65-year-old Caucasian male presents to clinic with symptoms of heart failure. In the past month he endorses NYHA class III symptoms and is not volume overloaded on physical exam. Medications include carvedilol 25mg twice daily, sacubitril 24mg/valsartan 26mg twice daily, spironolactone 25mg once daily, and torsemide 50mg once daily. Blood pressure is 128/74 mmHg and heart rate is 78 bpm. Labs are Na 142 mEq/L, K 3.8 mEq/L, SCr 1.1 mg/dL. Echocardiography reveals a left ventricular ejection fraction of 25%.

Increase carvedilol (1)

Increase sacubitril-valsartan (2)

Increase spironolactone (3)

Add dapagliflozin (4)

Do not make any of these medication changes (5)

Q9 A 38-year-old African American male presents to clinic with symptoms of heart failure. In the past month he endorses NYHA class II symptoms and is euvolemic on physical exam. Medications include carvedilol 37.5mg twice daily, lisinopril 30mg once daily, spironolactone

25mg once daily, and furosemide 40mg twice daily. Blood pressure is 122/84 mmHg and heart rate is 62 bpm. Labs are Na 131 mEq/L, K 4.2 mEq/L, Cr 1.5 mg/dL. Echocardiography reveals a left ventricular ejection fraction of 15%.

Add hydralazine and isosorbide dinitrate (1)

Increase lisinopril (2)

Add digoxin (3)

Switch lisinopril to sacubitril-valsartan (4)

Add dapagliflozin (5)

Do not make any of these medication changes (6)

Q10 A 73-year-old African American male presents to clinic with symptoms of heart failure. In the past month, he endorses NYHA class III symptoms and is not volume overloaded on physical exam. He also has a history of coronary artery disease. Medications include carvedilol 37.5mg twice daily, sacubitril-valsartan 49-51mg twice daily, spironolactone 25mg once daily, dapagliflozin 10mg daily, furosemide 60mg once daily, aspirin 81mg once daily, rosuvastatin 20mg once daily. Blood pressure is 122/84 mmHg and heart rate is 62 bpm. Labs are Na 136 mEq/L, K 4.1 mEq/L, Cr 1.6 mg/dL. Echocardiography reveals a left ventricular ejection fraction of 15%.

Increase furosemide (1)

Increase sacubitril-valsartan (2)

Increase spironolactone (3)

Add hydralazine and isosorbide dinitrate (4)

Do not make any of these medication changes (5)

Q11 A 60-year-old Asian male patient is referred for a new diagnosis of heart failure with reduced ejection fraction. In the past month, he endorses NYHA class II symptoms and is mildly volume overloaded on physical exam. He also has a past medical history of hypertension and type 2 diabetes. Medications include amlodipine 5mg once daily, metformin 1000mg twice daily, furosemide 40mg once daily, and atorvastatin 20mg once daily. Blood pressure is 134/76 mmHg and heart rate is 86 bpm. Labs are Na 132 mEq/L, K 4.2 mEq/L, Cr 1.5 mg/dL. Echocardiography reveals a left ventricular ejection fraction of 30%.

Add metoprolol succinate (1)

Add lisinopril (2)

Add spironolactone (3)

Increase amlodipine (4)

Do not make any of these medication changes (5)

Q12 A 64-year-old Caucasian male returns to clinic for follow up of his non-ischemic cardiomyopathy. In the past month, he endorses NYHA class II symptoms and is mildly volume overloaded on physical exam. Medications include carvedilol 12.5mg twice daily and furosemide 60mg once daily. Blood pressure is 122/74 mmHg and heart rate is 66 bpm. Labs are Na 137 mEq/L, K 4.2 mEq/L, Cr 1.4 mg/dL. Echocardiography reveals a left ventricular ejection fraction of 30%.

Add enalapril (1)

Increase carvedilol (2)

Add spironolactone (3)

Add dapagliflozin (4)

Do not make any of these medication changes (5)

Q13 A 48-year-old African American male returns to clinic for follow up of his heart failure with reduced ejection fraction. In the past month, he endorses NYHA class II symptoms and is not volume overloaded on physical exam. Medications include metoprolol succinate 200mg once daily, spironolactone 25mg once daily. He was previously on lisinopril but this was stopped due to cough. Blood pressure is 128/80 mmHg and heart rate is 72 bpm. Labs are Na 133 mEq/L, K 4.4 mEq/L, Cr 1.1 mg/dL. Echocardiography reveals a left ventricular ejection fraction of 20%.

Add sacubitril-valsartan (1)

Add valsartan (2)

Add ivabradine (3)

Add hydralazine and isosorbide dinitrate (4)

Do not make any of these medication changes (5)

Q14 A 76-year-old African American female returns to clinic for follow up of her nonischemic cardiomyopathy. In the past month, she endorses NYHA class III symptoms and is volume overloaded on physical exam. Medications include carvedilol 25mg twice daily and furosemide 80mg twice daily. Blood pressure is 140/78 mmHg and heart rate is 58 bpm. Labs are Na 140 mEq/L, K 5.0 mEq/L, SCr 2.4 mg/dL. Echocardiography reveals a left ventricular ejection fraction of 25%.

Add enalapril (1)

Add spironolactone (2)

Add hydralazine and isosorbide dinitrate (3)

Decrease carvedilol (4)

Do not make any of these medication changes (5)

Q15 A 55-year-old African American female presents to clinic with symptoms of heart failure. In the past month, she endorses NYHA class III symptoms and is not volume overloaded on physical exam. She also has a past medical history of coronary artery disease and hypertension. Medications include metoprolol succinate 200mg once daily, lisinopril 20mg once daily, spironolactone 25mg once daily, dapagliflozin 10mg daily, bumetanide 2mg once daily, aspirin 81mg once daily, and atorvastatin 40mg once daily. Blood pressure is 98/68 mmHg and heart rate is 80 bpm. Labs are Na 143 mEq/L, K 3.7 mEq/L, SCr 1.1 mg/dL. Echocardiography reveals a left ventricular ejection fraction of 30%.

Add digoxin (1)

Add ivabradine (2)

Switch lisinopril to sacubitril-valsartan (3)

Add hydralazine and isosorbide dinitrate (4)

Do not make any of these medication changes (5)

Q16 A 62-year-old African American male presents to clinic with symptoms of heart failure. In the past month, he endorses NYHA class III symptoms and is not volume overloaded on physical exam. He also has a history of type 2 diabetes and hypertension. Medications include carvedilol 6.25mg twice daily, lisinopril 5mg once daily, spironolactone 25mg once daily, digoxin 125mcg once daily, furosemide 60mg twice daily, dapagliflozin 10mg daily, metformin 500mg twice daily. Blood pressure is 90/62 mmHg and heart rate is 80 bpm. Labs are Na 143 mEq/L, K 3.7 mEq/L, SCr 1.1 mg/dL. Echocardiography reveals a left ventricular ejection fraction of 30%.

Increase lisinopril (1)

Add ivabradine (2)

Increase carvedilol (3)

Add hydralazine and isosorbide dinitrate (4)

Do not make any of these medication changes (5)

Q17 A 50-year-old African American presents to clinic with symptoms of heart failure. In the past month, she endorses NYHA class III symptoms and is not volume overloaded on physical exam. She also has a past medical history atrial fibrillation and type 2 diabetes. Medications include carvedilol 6.25mg twice daily, lisinopril 5mg once daily, spironolactone 25mg once daily, furosemide 40mg twice daily, dapagliflozin 10mg daily, apixaban 5mg twice daily, and metformin 500mg twice daily. Blood pressure is 90/62 mmHg and heart rate is 72 bpm. Labs are Na 132 mEq/L, K 4.2 mEq/L, SCr 1.2 mg/dL. Echocardiography reveals a left ventricular ejection fraction of 15%.

Add digoxin (1)

Add ivabradine (2)

Increase carvedilol (3)

Increase lisinopril (4)

Do not make any of these medication changes (5)

Q18 A 47-year-old African American male presents to clinic with a recent diagnosis of heart failure. In the past month he endorses NYHA class II symptoms and is not volume overloaded on physical exam. He also has a history of hypertension. Medications include amlodipine 5mg once daily and furosemide 20mg once daily. Blood pressure is 138/90 mmHg and heart rate is 78 bpm. Labs are Na 142 mEq/L, K 3.8 mEq/L, Cr 1.1 mg/dL. Echocardiography reveals a left ventricular ejection fraction of 25%.

Add lisinopril (1)

Add carvedilol (2)

Add spironolactone (3)

Increase amlodipine (4)

Do not make any of these medication changes (5)

Q19 A 66-year-old Caucasian female returns to clinic for follow up of her nonischemic cardiomyopathy. In the past month, she endorses NYHA class III symptoms and is not volume overloaded on physical exam. She also has a past medical history of chronic kidney disease and hypertension. Medications include carvedilol 6.25mg twice daily, lisinopril 10mg once daily and furosemide 80mg twice daily. Blood pressure is 114/78 mmHg and heart rate is 62 bpm. Labs are Na 140 mEq/L, K 5.0 mEq/L, Cr 1.8 mg/dL. Echocardiography reveals a left ventricular ejection fraction of 25%.

Increase lisinopril (1)

Add spironolactone (2)

Increase carvedilol (3)

Add hydralazine and isosorbide dinitrate (4)

Do not make any of these medication changes (5)

Q20 A 54-year-old Caucasian male returns to clinic for follow up of his nonischemic cardiomyopathy. In the past month, he endorses NYHA class II symptoms and is not volume overloaded on physical exam. He also has a past medical history of chronic kidney disease and hypertension. Medications include carvedilol 6.25mg twice daily, lisinopril 10mg once daily, and furosemide 60mg twice daily. Blood pressure is 108/74 mmHg and heart rate is 80 bpm. Labs are Na 142 mEq/L, K 4.8 mEq/L, Cr 1.9 mg/dL. Echocardiography reveals a left ventricular ejection fraction of 25%.

Increase lisinopril (1)

Add spironolactone (2)

Increase carvedilol (3)

Add ivabradine (4)

Add dapagliflozin (5)

Do not make any of these medication changes (6)
