# Supplementary figures and images for: Differences in provider approach to initiating and titrating guideline directed medical therapy in heart failure with reduced ejection fraction
Source: BMC Cardiovasc Disord. 2024 May 11;24:247. doi: 10.1186/s12872-024-03911-1 (PMC11087241; doi:10.1186/s12872-024-03911-1)

Figure S1. Geographic distribution of survey respondents.

Respondent Location

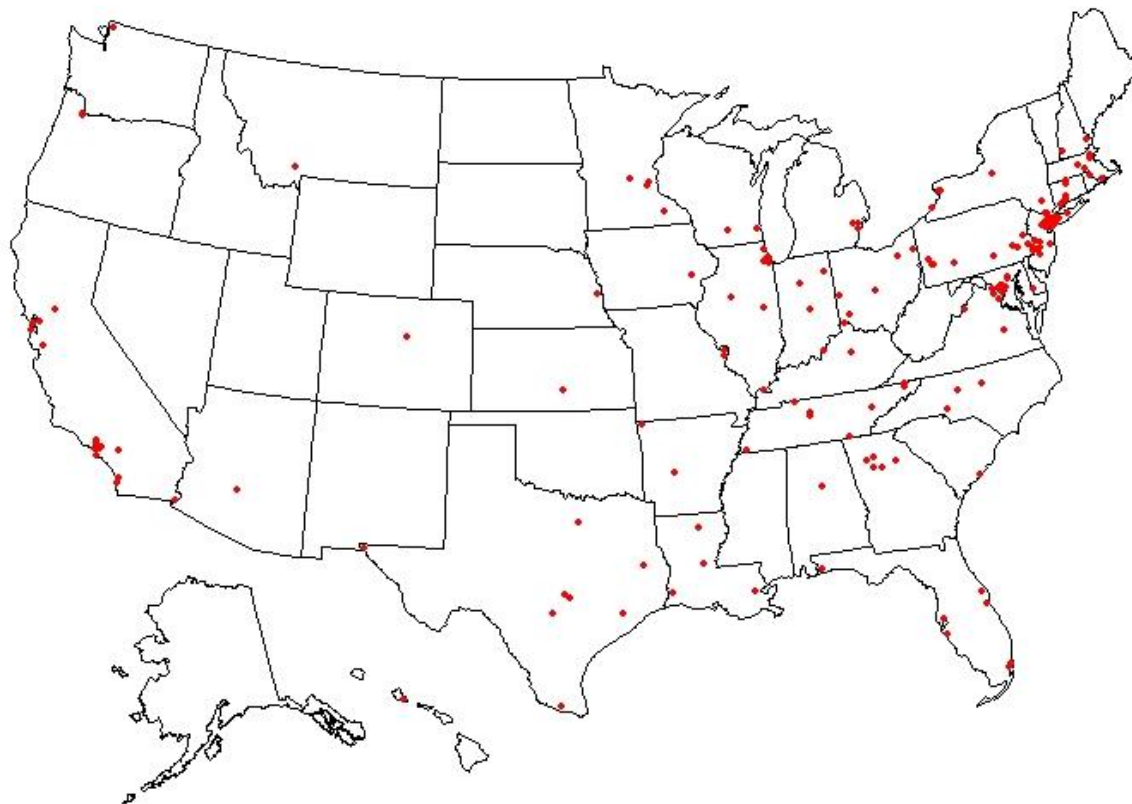

Supplement: Supplementary file 2 — Supplementary Material 2 [file 12872_2024_3911_MOESM2_ESM.pdf]
