# Supplementary material for: Differences in provider approach to initiating and titrating guideline directed medical therapy in heart failure with reduced ejection fraction
Source: BMC Cardiovasc Disord. 2024 May 11;24:247. doi: 10.1186/s12872-024-03911-1 (PMC11087241; doi:10.1186/s12872-024-03911-1)
Supplement: Supplementary file 3 — Supplementary Material 3 [file 12872_2024_3911_MOESM3_ESM.pdf]

Table S1. Categories for each survey question.

	**Medication Naive**	**Titrate GDMT**	**ACEi/ARB initiation**	**MRA initiation**	**Entresto switch/initiation**	**HYD/ISDN initiation**	**SGLT2i initiation**	**Digoxin initiation**	**Ivabradine initiation**	**CKD patients**	**Borderline hemodynamics**
**1**				X	X						
**2**		X			X	X					
**3**				X			X				
**4**		X									
**5**		X			X						
**6**		X					X				
**7**					X		X				
**8**		X					X				
**9**		X			X		X				
**10**		X				X					
**11**	X										
**12**			X				X				
**13**			X		X						
**14**						X				X	
**15**								X	X		X
**16**		X							X		X
**17**								X	X		X
**18**	X										
**19**		X								X	
**20**		X		X			X			X	
